# Comment on Anwer et al. Rehabilitation of Upper Limb Motor Impairment in Stroke: A Narrative Review on the Prevalence, Risk Factors, and Economic Statistics of Stroke and State of the Art Therapies. *Healthcare* 2022, *10*, 190

**DOI:** 10.3390/healthcare10050846

**Published:** 2022-05-05

**Authors:** Giovanni Morone, Daniele Giansanti

**Affiliations:** 1 Department of Life, Health and Environmental Sciences, University of L’Aquila, 67100 L’Aquila, Italy; giovanni.morone@univaq.it; 2 Centre Tisp, The Italian National Institute of Health, 00161 Rome, Italy

We are writing to you as the corresponding author of the interesting review study entitled “Rehabilitation of Upper Limb Motor Impairment in Stroke: A Narrative Review on the Prevalence, Risk Factors, and Economic Statistics of Stroke and State of the Art Therapies” [1].

We found that this work is particularly stimulating and provides a great added value to the field.

Specifically, we believe that this review has the great merit of focusing on both key aspects of the integration of upper limb rehabilitation in the *health domain* and aspects relating to technological innovation, including, in addition to purely clinical aspects, economic aspects and risk factors.

In the Special Issue (SI) [2,3] “*Rehabilitation and Robotics: Are They Working Well Together*?” we addressed these issues with reference to the use of robotic technologies. In particular, we focused on clinical studies on the use of robotic technologies in the rehabilitation field, ranging from the field of disabling pathologies of neurological origin to the field of injuries, also including the support of the elderly (in particular, frail persons) or of people with communication disabilities. It must be borne in mind that, in the robotics sector, despite major developments there is no uniformity or standardization of use. Robotics is often used on a very limited basis to pilot and/or research projects. The purpose of the Special Issue was to take stock of the issues that hinder the integration of robotics in clinical practice and on useful initiatives in this direction. We consider your study to be very important for having faced, along with many other themes, the theme of the robotics. Your analytical study reviewed different technologies used for therapies such as functional electric stimulation, noninvasive brain stimulation including transcranial direct current stimulation, transcranial magnetic stimulation, invasive epidural cortical stimulation, virtual reality rehabilitation, robot-assisted training, and telerehabilitation. These technologies can be used alone or even in synergy. As an example, robotics is currently also used in telerehabilitation.

The review highlighted, in line with the SI [2,3], both the potential of robotics in perspective and its limits.

Among potential applications, it was highlighted how:

- Pilot studies [4] have shown promisingly positive results of robot-assisted rehabilitation for recovery and plasticity following a stroke.
- Assistive technologies (robotic prosthetic limbs and devices) are useful and promising for supporting the human body’s lost function [5].

The limits and perplexities of the effectiveness of the use of robotics in comparison with other traditional therapies were also highlighted. Some included studies reported that such comparisons in some applications:

- Were positive but not satisfactory [6].
- Did not reveal a significant improvement of upper limb functionalities [7,8,9,10,11].

Robotics as a single technology or integrated with other different biomedical technologies [1], ranging from functional electric stimulation to telerehabilitation, represents an important perspective of research in this field for scholars.

In this same field, very recent studies [12,13,14] have addressed the potential of technologies based on artificial intelligence (AI) in neurological rehabilitation applications based on robotics. AI looks promising for both face-to-face rehabilitation [12,13] and remote activities [14].

The study by Yang et al. [12] described how the rapid development of intelligent computing has attracted the attention of researchers of robotic neurorehabilitation with computational intelligence, reporting that Artificial Intelligence affected both the mechanical structures and the control methods in rehabilitation robotics.

The study by Nizamiz et al. [13] pointed out how novel, wearable robotic devices are being tailored to specific patient populations, such as those with traumatic brain injury, stroke, and amputation, and how AI could facilitate the developments in robot-assisted rehabilitation in motor learning and in generating movement repetitions by decoding the brain activity of patients during therapy.

The study by Lambercy et al. [14] faced the perspective of robot-assisted therapy in a minimally supervised and decentralized manner, using rehabilitation devices that are portable, scalable, and equipped with clinical intelligence, remote monitoring, and coaching capabilities.

Considering the research you have undertaken, we would like to hear your opinion about that, and, in particular, if you think that among current and future developments, AI will play an important role in this sector in an autonomous contribution and/or in support of the technologies mentioned in your review study.

We would strongly appreciate an opinion on this as a reply in the SI.

## Data Availability

Not applicable.
